# Factors that can minimize bleeding complications after renal biopsy

**DOI:** 10.1007/s11255-013-0560-6

**Published:** 2014-08-24

**Authors:** M. S. Zhu, J. Z. Chen, A. P. Xu

**Affiliations:** Department of Nephrology, Sun Yat-Sen Memorial Hospital of Sun Yat-Sen University, Guangzhou, 510120 People’s Republic of China

**Keywords:** Renal biopsy, Bleeding complication, Risk factors, Measures

## Abstract

Renal biopsy is a very important diagnostic tool in the evaluation of renal diseases. However, bleeding remains to be one of the most serious complications in this procedure. Many new techniques have been improved to make it safer. The risk factors and predictors of bleeding after percutaneous renal biopsy have been extensively reported in many literatures, and generally speaking, the common risk factors for renal biopsy complications focus on hypertension, high serum creatinine, bleeding diatheses, amyloidosis, advanced age, gender and so on. Our primary purpose of this review is to summarize current measures in recent years literature aiming at minimizing the bleeding complication after the renal biopsy, including the drug application before and after renal biopsy, operation details in percutaneous renal biopsies, nursing and close monitoring after the biopsy and other kinds of biopsy methods.

## Introduction

Since the first successful percutaneous renal biopsy in 1950s, renal biopsy has been an essential procedure in establishing the histological diagnosis, adequate therapy and prognosis of kidney diseases [1–4]. With the help of ultrasound guidance and spring-loaded biopsy needle, this procedure has became safer, but like many invasive procedures, renal biopsy accompanies with the risk of potential complications such as bleeding, infection, pain, loss of kidney and even death, while bleeding complications are the most common one. Post-renal biopsy bleeding complications are classified as either major or minor. Major complications include hemorrhage requiring transfusion, bleeding with necessity of nephrectomy and death. The frequency of major hemorrhage has ranged from 0 to 6 % in different studies [1, 5, 6]. Minor complications are defined as gross hematuria or subcapsular perinephric hematoma which will spontaneously be resolved without need for further intervention. For example, according to the survey of Shidham et al. [1], the incidence of post-renal biopsy bleeding contains the following: gross hematuria, 1.9 %; hematoma, 0.9 %; and blood transfusion, 2.5 %. Also, the meta-analysis by Corapi et al. [7] summaries that the complication rate of macroscopic hematuria is 3.5 % (95 % CI 2.2–5.1 %), and erythrocyte transfusion is 0.9 % (95 % CI 0.4–1.5 %). Besides, in the studies by Daram et al. [8], around a quarter of patients had >10 % decline in hematocrit at 24 h, requiring blood transfusion in 3.6–9 % of the patients and an incidence of gross hematuria of 3.6–17 %. A large series of >9,000 biopsies in pediatric and adult patients is recently reported by Tondel et al. [9], and their study shows that gross hematuria appears after biopsy in 1.9 % of the patients and 0.9 % of patients need blood transfusion. The frequencies are 1.9 and 0.9 % in adults and 1.7 and 0.1 % in children. All of these studies show that though renal biopsy has became safer, it is still not without risk.

## Risk factors on post-biopsy bleeding

To determine the effect of various risk factors on post-biopsy bleeding, many retrospective cohort studies have been performed. Generally speaking, the common risk factors for renal biopsy complications focus on hypertension, high serum creatinine, bleeding diatheses, amyloidosis, advanced age, gender and so on, though some factors are still under debate [1, 2, 7–11]. Among the many risk factors, parts of them are modifiable, such as hypertension and bleeding diatheses, which can be modified by corresponding drugs. So the more we know about the risk factors for bleeding complications of renal biopsy, the better measures we can take to reduce the complication rates.

### Hypertension

Blood pressure was significantly higher in patients with complications, and it was regarded as a risk factor for bleeding complications independently of all other variables by logistic multivariate analysis [25]. The study of Shidham et al. [1] showed that the risk of bleeding increased with high systolic blood pressure (SBP), diastolic blood pressure (DBP) or mean arterial pressure (MAP). For example, the risk of bleeding was 10.7 % when SBP >160 mmHg compared with 5.3 % when the SBP <160 mmHg (*P* = 0.03). Similarly, the risk was 12.5 % when the MAP >120 mmHg compared with 5.1 % when the MAP <120 mmHg (*P* = 0.009).

### High serum creatinine

The serum creatinine also affected the bleeding complications after renal biopsy. Patients with major complications had higher serum creatinine levels when compared with those who did not. In the study of Whittier et al. [10], multivariate analysis using logistic regression showed that serum creatinine at baseline predictive of a complication and patients with a serum creatinine >5.0 mg/dl were 2.3 times as likely to have a complication (odds ratio, 2.3; 95 % CI 1.3–4.1; *P* < 0.005). In another study, for patients with creatinine >2 mg/dl, the risk ratio for post-biopsy bleeding (PBB) was 5.89 when compared with patients with creatinine <2 mg/dl, and logistic regression analysis showed that serum creatinine of >2.0 mg/dl was associated with an odds ratio of 2.56 (CI 1.48–4.42, *P* = 0.001) for PBB [1]. All these similar results showed that the risk of bleeding postoperatively increases with worsening levels of renal insufficiency.

### Prolonged bleeding time

The role of prolonged bleeding time was controversial. Previous studies on renal biopsy did not demonstrate a firm relationship between the bleeding time and bleeding complications. Eiro et al. [25] performed a research that pro-thrombin time, activated partial thromboplastin time and bleeding time were measured routinely in all patients before performing renal biopsy, and patients who had at least one abnormal value were regarded as contraindicated. In the study of Manno et al. [31], baseline partial thromboplastin time was significantly higher in patients who developed post-biopsy bleeding complications compared with those who did not (102.7 ± 11.8 vs. 100.1 ± 10.0 %, *P* = 0.013). However, in the study by Mackinnon et al. [12], the correction of prolonged bleeding time with pro-coagulants might not significantly reduce the risk of clinically important bleeding and they warned us that the administration of a pro-coagulant might increase the risk of a thrombotic vascular event, and thus, the practice of administering pro-coagulants routinely to correct a prolonged bleeding time should be reassessed. In the study of van den Hoogen et al. [13], the platelet function analyzer (PFA) had a higher positive and similar negative predictive value compared to the bleeding time. When a screening of the primary hemostasis was performed prior to a renal biopsy, they recommended using the PFA instead of the bleeding time (BT).

### Histological diagnosis and amyloidosis

The relationship between the histological diagnosis and the complications of post-renal biopsy had been studied. Fisi et al. [14] performed a research which showed that in patients with the diagnosis of diabetic nephropathy or acute tubular necrosis, the overall complication prevalence was significantly lower compared to others, while complication rate was the highest in patients who suffered from thin basement membrane syndrome, vasculitis, rapidly progressive glomerulonephritis (RPGN) or acute interstitial nephritis. However, in the latter patients, the higher rate of complications may be related to the possibility of low glomerular filtration rate (GFR) because of acute kidney injury in many of these instances. In the meta-analysis by Corapi et al. [7], acute kidney injury was related to significantly higher rates of need for transfusion after renal biopsy (1.1 vs. 0.04 %; *P* < 0.001). The result of another study by Tondel et al. [9] also showed that low estimated GFR (estimated GFR = 30–59 ml/min per 1.73 m^2^ [OR = 4.90 (1.60–14.00)] and estimated GFR <30 ml/min per 1.73 m^2^ [OR 15.50 (5.60–43.00)]) had higher odds in adjusted analysis for risk factors for major complication after renal biopsy. So perhaps the low GFR was a confounding factor which would make it look like that certain diseases were linked with higher complication rates. Still, in the latter patients with a higher risk, stricter post-biopsy monitoring might be necessary. Amyloidosis was also regarded as an important risk factor for bleeding complication after renal biopsy in some studies [25]. To assess its risk, Soares et al. [15] performed a large retrospective study and their result showed that incidences of bleeding after kidney biopsy were similar between the amyloidosis and control groups (9.9 vs. 10.6 %). They suggested that the doctrine that patients with amyloid were at a greater risk of hemorrhage after kidney biopsy appeared to be the result of reporting bias. In fact, since patients with any systemic amyloidosis could have widespread small-vessel fragility by direct vessel infiltration and acquired hemostatic abnormalities caused by coagulation factor deficiency, it was reasonable for us to undertake the procedure in such patients with great care. So it was reasonable for us to check the hemostatic condition and whether hypertension existed in the patients with amyloidosis before the renal biopsy was done.

### Age and gender

Other risk factors included that there was a significantly greater proportion of women compared with men who developed post-biopsy bleeding complications. In the study by Manno et al. [31], the increased risk of post-biopsy bleeding in women might be explained by their different body composition. The greater percentage of fat mass in women might be responsible for a tendency for hematoma to expand in the peri-renal fatty tissue. There was also a variable distribution in the risk of post-biopsy bleeding according to age, as the incidence of bleeding complication was more in older patients than in younger ones [16, 25]. A hypothesis was made in the study by Kohli et al. [16] that the higher incidence of gross hematuria in the elderly might have been due to the arterial wall changes related to aging. Due to the near ubiquitous presence of arteriosclerosis in the elderly, the ability of severed small vessels to undergo vasoconstriction might be impaired resulting in hematuria. Also, in the study of Whittier et al. [10], patients with a major complication after biopsy were older (53 ± 17 vs. 43 ± 18 years; *P* < 0.05).

## Measures that related to minimizing the bleeding complications

Despite the understanding of predisposing risk factors, there is still no definitive way to predict whether one patient will surely develop a serious complication. In our review of the literature, we focus on the possible ways which are in an attempt to minimize the bleeding complication in post-renal biopsy.

### Application of drugs before and after renal biopsy

#### Antihypertensive drugs

Many studies showed that the hypertension was one of the important risk factors for post-renal biopsy bleeding, and thus, controlling the hypertension might help to reduce its incidence. In order to get hypertension under control before the biopsy, a calcium channel blocker or nifedipine was administered prior to renal biopsy to patients with a blood pressure reading >140/90 mmHg [25, 31]. For example, Maya et al. [5] performed a research that patients with uncontrolled hypertension (>160/100 mmHg) were treated with oral clonidine (0.1 mg) or intravenous hydralazine (10 mg) to lower their blood pressure prior to the biopsy. But we should also be cautious that acutely lowering blood pressure before biopsy using dihydropyridine type calcium channel blockers had been associated with an increased risk of bleeding due to vasodilatation and inhibition of platelet function [16]. However, there have been no systematic studies in renal biopsy to address this issue.

#### Desmopressin acetate

Desmopressin has a long history of being used to decrease the prolonged bleeding time in patients with uremia in an effort to improve hemostasis. It has also been used in high-risk patients undergoing kidney biopsy [2, 3, 13, 17]. Manno et al. [18] performed a study to evaluate the effect of pre-biopsy administration of desmopressin acetate versus placebo in the incidence of post-biopsy bleeding complications. In their study patients who received desmopressin acetate had significantly decreased the risk of post-biopsy bleeding (13.7 vs. 30.5 %; *P* = 0.01), and in addition, the size of the hematoma, if present, was on average smaller in the intervention group (median, 208 vs. 380 mm^2^; *P* = 0.006). Still, this was a single-center design study which decreased the generalization of the results and its external validity. In their study, desmopressin acetate was used for all patients regardless of GFR, whereas in clinical practice, it was probably only used in patients with significantly deteriorated GFR. More studies are needed to confirm the effect of pre-biopsy treatment with desmopressin acetate.

#### Recombinant activated factor VII

In the case report of Maksimovic et al. [19], recombinant activated factor VII (rFVIIa) was used to treat uncontrolled bleeding which was caused by renal biopsy after unsuccessful treatment with desmopressin. With the application of rFVIIa, the bleeding stopped immediately. Thus, a conclusion was made that rFVIIa appeared efficacious and well tolerated in the treatment of post-biopsy bleeding in a kidney transplant patient with renal failure. This case report indicates the effectiveness of rFVIIa in the treatment of uremic patients, though more prospective studies of its efficacy and safety in such patients are needed.

#### Preventative use of drugs

The preventative use of hemostatic agents in patients without any coagulation disorder before renal biopsy is common in some Chinese hospitals, though it may not often be seen in other countries. The common drugs used are Reptilase and vitamin K1. After being injected intravenously, Reptilase can divide the α-subunit of fibrinogen into A-peptide and fibrin monomers, the later of which polymerizes and helps improve hemostasis. Feng HL [20] reported in his study that before the renal biopsy, 1KU Reptilase given to the patients by intravenous injection could effectively reduce the bleeding compared with the control group (hemoglobin reduction: control group 16 ± 9 g/L vs. treatment group 5 ± 3 g/L; *P* < 0.05). Similar results can be seen in the study of Zhou [21], which shows that vitamin K1 10 mg used 3 days and Reptilase 1KU used 1 h before and after renal biopsy could effectively reduce the incidence rate of bleeding complication after the renal biopsy procedures (gross hematuria: control group 7.5 % vs. treatment group 2.5 %; *P* < 0.05). Though their study results need further confirmation, it presents a possible approach to deal with the commonly seen bleeding complication after the renal biopsy.

#### Antiplatelet agents

Generally speaking, the antiplatelet agents and non-steroidal anti-inflammatory drugs were discontinued 7 days before the biopsy, which could be seen in a series of retrospective cohort studies [3–5, 14, 16]. For example, the mean duration of antiplatelet agent cessation was 7.9 ± 2.2 days before and 6.0 ± 3.4 days after renal biopsy in a French nationwide study [17]. However, in the study by Mackinnon et al. [12], the ongoing use of antiplatelet agents was not associated with an increase in the risk of clinically significant bleeding complications and the withdrawal of antiplatelet agents had a risk of causing coronary syndrome. They performed a retrospective study of 1120 biopsies to define whether it was necessary to stop antiplatelet agents, and their result showed that stopping antiplatelet agents before biopsy was associated with a lower rate of minor complications (31.0 vs. 11.7 %; *P* = 0.008), but there was no difference in the rate of major complications. Atwell et al. [22] also performed a research about the influence of aspirin on the biopsy, and their result showed that the incidence of bleeding in patients taking aspirin within 10 days before biopsy was 0.6 % (18/3,195), which was not statistically different compared with the incidence of bleeding in those not taking aspirin (52/11,986, 0.4 %; *P* = 0.34). Interestingly, a meta-analysis of the literature related to peri-procedural aspirin use proved that an approximately 50 % increase in the bleeding rate in those taking aspirin at the time of surgery or biopsy [23]. Besides, the platelet function analyzer was recommended to be used in the screening of primary hemostasis instead of bleeding time [13]. In fact, whether the antiplatelet agents and non-steroidal anti-inflammatory drugs should be discontinued or not depend on the condition of the patients. For those without heart disease, it is a better choice for them to stop antiplatelet agents before the biopsy, while for those with high risk of coronary syndrome, we need to balance the risks and benefits of performing biopsy, bleeding complication and acute coronary syndrome.

### Operation details in percutaneous renal biopsies

Post-biopsy complications may be even less frequent with the use of smaller gauge needles. In the study of Kim et al. [24], the use of 14-gauge versus 18-gauge in native kidney renal biopsies had been compared in a randomized trial that showed a greater incidence of complications in manual biopsy with 14-gauge needle compared with automated renal biopsy using 18-gauge guns. Though their study had been flawed as it may reflect the impact of biopsy technique rather than needle size, similar results could be seen in the meta-analysis of Corapi et al. [7], which was that significantly higher rates of transfusion after renal biopsy were related to the needle size: 14-gauge compared with smaller needles (2.1 % vs. 0.5 %; *P* = 0.009). The study of Tondel et al. [9] showed that the dominance o f 16- and 18-G needles may result in higher focus on minimizing risk factors and the acceptance of less tissue per needle pass. In their study, the median number of glomeruli per subject was comparable with other studies, while the percentage of biopsies characterized as representative tissue was in the same level irrespective of needle size.

The study of Eiro et al. [25] showed that the frequency of the puncture was not significantly different between moderate complications and no or mild complications; however, by logistic multivariate analysis, the frequency of the puncture was an independent risk factor. The relationship between the bleeding complications and the depth of needle insertion was studied. Pasquariello et al. [26] performed a research that if the trigger was pushed exactly at the depth previously calculated by a mathematical formula: BW/H less 0.5 (body weight expressed in hectograms divided by patient height expressed in centimeters), it would be extremely useful to reduce the incidence of bleeding complications and allowed an adequate sampling for diagnostic evaluation in all cases. Another study also found some positive relationship between hemoglobin decrease and depth of needle insertion and warned that nephrologists should be cautious of depth of needle insertion to avoid major hemorrhage complication [4].

Some studies showed that there was no inevitable relationship between the experience of the operator and the incidence of post-biopsy bleeding. In the study of Maya et al. [5], the complications rate was low despite their performance by first-year nephrology fellows. The reason may be that the use of real-time ultrasound guidance permitted 91 % of the patients to require just one or two needle passes, which likely contributed to the low major complication rate. Similar results could be seen in another study, which showed that the use of the real-time ultrasound-guided technique minimized the risk of major complications even in the hands of inexperienced operators [27]. In summary, the real-time ultrasound guidance makes this operation safer.

### Nursing and close monitoring after the biopsy

Fisi et al. [14] in their study showed that after the renal biopsy, patients remaining in bed lying on their backs all the time with a sandbag under the site of puncture for 4 h was a way to reduce the complications and a fluid intake of at least 3L was recommended in this period. In fact, this was also a common measure taken to deal with post-renal biopsy complications in some Chinese hospitals, for example, a sandbag or abdominal compression belts were used to press the puncture site for 6 h with blood pressure monitoring and the urinary catheter might be inserted in order to reduce the patients’ movements, which may cause bleeding [21, 28, 29]. Few studies showed the influence of such procedure on the incidence rate of post-renal biopsy bleeding; however, it had been debated that since kidney was deep in the body and the pressure of the sandbag worked on the surface of the back, maybe the hemostatic effect would not be as perfect as imagine. Besides, the sandbag would cause discomfort when placed on the back of the patients on bed. In these aspects, the effectiveness of this method and its application needs more clinical observations.

Whittier et al. [10] performed a study on the timing of the complications and showed that complications happened in 67 % patients in 8 h, and in 89 % patients at ≤24 h, which meant an observation time of up to 24 h was optimal because an observation period of <8 h meant missing about 22 % of complications. In another research, if the patient showed no signs of bleeding complications 18 h after the biopsy, there was little probability for them to grow thereafter [25]. These results presented the importance of the observation of monitoring after the biopsy. As the utility of early post-renal biopsy ultrasound was useful in predicting the risk of major bleeding complications, some studies showed that the ultrasound findings 1 h post-biopsy were clinically helpful in predicting the bleeding complication. While the presence of a hematoma at 1 h post-biopsy was not predictive of a complicated post-biopsy course, the absence of a hematoma 1 h post-biopsy was highly predictive of an uncomplicated post-biopsy course [2]. In the retrospective study by Ishikawa et al. [4], perirenal hematoma >2 cm immediately after biopsy was the strongest predictor of more severe anemia the morning after biopsy. Their findings were similar with other reports of progressive anemia with larger post-biopsy hematoma [30]. This indicated that ultrasonographic evaluation of hematoma size immediately after renal biopsy was useful in predicting potentially severe blood loss. A conclusion was also made in the study by Daram et al. [8] that the degree of decline in Hct at 6 h was predictive of the degree of decline at 18–24 h. But this result still needs to be validated in larger prospective studies.

### Other kinds of biopsy methods

With the new technologies of real-time ultrasonography for guiding the procedure and the use of automatic biopsy needles, percutaneous renal biopsies are well established as a safe and effective technique for obtaining samples of renal parenchyma, and it has improved the rate of successful diagnosis in over 95 % of cases [32]. However, absolute and relative contraindications for the percutaneous approach do exist. When renal histology is necessary for clinical management but percutaneous biopsy is contraindicated or unsuccessful, other methods of renal biopsy by experienced physicians may be attempted.

#### Transjugular renal biopsy

A study was performed to describe the indications for transjugular renal biopsy (TJRB) and the results showed that the most common indication was a bleeding diathesis due to thrombocytopenia or coagulopathy, and other recognized indications for a TJRB include inability to cooperate with the percutaneous procedure, severe hypertension, a solitary or horseshoe kidney, end-stage renal disease or bilaterally small kidneys and morbid obesity [33]. In a large study by Cluzel et al. [34], TJRB was compared with percutaneous renal biopsies and the result showed that there was no difference in the diagnostic yield or in complication rates. Major complications occurred in ~1 % when using both routes. The number of glomeruli per biopsy was smaller using the TJRB route (11.2 vs. 9.8; *P* = 0.361). This reduction in yield probably resulted from the smaller needle size used for TJRB. Levi et al. [35] performed a study that their initial experience with TJRB was similar or better than in prior reports with regard to both diagnostic yield and complication rates. So TJRB is recognized as an alternative, safe and effective technique in patients with renal parenchymal disease. However, due to its technical complexity and the smaller amount of glomeruli retrieved when compared to percutaneous biopsy, it should be reserved for high-risk patients.

#### Open renal biopsy

Alternative methods except transjugular renal biopsy have also been attempted for obtaining samples of kidney tissue samples in patients with contraindications for the percutaneous approach. The open renal biopsy (surgical approach) has been established as a safe and effective technique for obtaining renal tissue. Multiple bilateral renal cysts are a relative contraindication for the percutaneous approach due to the risk of complications and the difficulty in obtaining adequate tissue samples. In these situations, an open renal biopsy through a posterior or flank incision is a viable option. However, both the risk of general anesthesia and the delayed recovery time associated with the open approach are of obvious concern [32, 36].

#### Transurethral renal biopsy

There are other less invasive alternatives. A special method of renal biopsy, the transurethral approach, had been described [37]. In this case report, a 62-year-old woman underwent the procedure with an 18-gauge needle via cystoscopy and 28 glomeruli were retrieved. The patient was observed for 24 h after the procedure and had a small subcapsular hematoma when checked by abdominal CT on routine evaluation. However, the transurethral approach is seldom mentioned afterward.

#### Laparoscopic renal biopsy

Laparoscopic renal biopsies can be performed using a retroperitoneal or transperitoneal approach. These biopsy methods allow for identification of the kidney, and the biopsy and hemostasis can be performed under direct visualization. Additionally, it is minimally invasive with very short patient recovery and convalescence times in the majority of cases. The retroperitoneoscopy or transperitoneal renal biopsy is in fact that currently recommended procedure for pediatric cases. Recently, a technique has been proposed that combines the laparoscopic approach with a percutaneous needle biopsy. This approach combines the advantages of the percutaneous biopsy with the minimal trauma and low morbidity associated with laparoscopic procedures [36].

## Conclusions

The renal biopsy is a very important and useful diagnostic tool for kidney diseases, and with the development of new technique, it has become much safer, but the bleeding complication does exist. There are many factors that may affect the post-biopsy complications, and thus, careful observation in the hospital is required for some patients with high risk. Among the many risk factors, parts of them are modifiable, so the medical data of patients should be checked whether the above risk factors such as hypertension, abnormality of the coagulation, high serum creatinine exist and measures should be taken to get the conditions under control if necessary. Before the renal biopsy, whether the preventive use of desmopressin acetate or other hemostatic agents should be adopted depends on the exact effect of it and needs further clinical trials. Also, whether the antiplatelet agents and non-steroidal anti-inflammatory drugs should be discontinued or not depends on the condition of the patients. For those without heart disease, it is advisable to stop antiplatelet agents for at least 1 week before the biopsy; however, for those with high risk of coronary syndrome, we need further risk benefit analysis. Whenever possible the biopsy procedure should be performed under real-time ultrasound or CT scan guidance and via an automated spring-loaded biopsy gun, rather than performed blindly. We should be cautious about the depth of the needle insertion as to get enough tissue for histological diagnosis and to avoid much traumatic damage to the renal parenchyma whose blood flow is abundant. Besides, the close monitoring of the patient in the post-biopsy period is of great importance. We believe that with the understanding of the risk factors and the considerate measures taken, and the use of real-time imaging and automated gun biopsy needles, the renal biopsy has become a safe procedure today.
